# Do adolescents always take more risks than adults? A within-subjects developmental study of context effects on decision making and processing

**DOI:** 10.1371/journal.pone.0255102

**Published:** 2021-08-02

**Authors:** Gail M. Rosenbaum, Vinod Venkatraman, Laurence Steinberg, Jason M. Chein

**Affiliations:** 1 Department of Psychology, Temple University, Philadelphia, Pennsylvania, United States of America; 2 Department of Psychology, New York University, New York, New York, United States of America; 3 Department of Marketing and Supply Chain Management, Temple University, Philadelphia, Pennsylvania, United States of America; Texas A&M University, UNITED STATES

## Abstract

Adolescents take more risks than adults in the real world, but laboratory experiments do not consistently demonstrate this pattern. In the current study, we examine the possibility that age differences in decision making vary as a function of the nature of the task (e.g., how information about risk is learned) and contextual features of choices (e.g., the relative favorability of choice outcomes), due to age differences in psychological constructs and physiological processes related to choice (e.g., weighting of rare probabilities, sensitivity to expected value, sampling, pupil dilation). Adolescents and adults made the same 24 choices between risky and safe options twice: once based on descriptions of each option, and once based on experience gained from sampling the options repeatedly. We systematically varied contextual features of options, facilitating a fine-grained analysis of age differences in response to these features. Eye-tracking and experience-sampling measures allowed tests of age differences in predecisional processes. Results in adolescent and adult participants were similar in several respects, including mean risk-taking rates and eye-gaze patterns. However, adolescents’ and adults’ choice behavior and process measures varied as a function of decision context. Surprisingly, age differences were most pronounced in description, with only marginal differences in experience. Results suggest that probability weighting, expected-value sensitivity, experience sampling and pupil dilation patterns may change with age. Overall, results are consistent with the notion that adolescents are more prone than adults to take risks when faced with unlikely but costly negative outcomes, and broadly point to complex interactions between multiple psychological constructs that develop across adolescence.

## Introduction

During adolescence, increased autonomy confers opportunities for independent decision making. Although adolescents sometimes make adult-like choices, teens are often confronted with risky choices where their actions can lead to consequential outcomes (e.g., whether to take drugs at a party, whether to get in a car with a driver who may have been drinking). In such real-world contexts, heightened adolescent risk taking is well documented [1,2]. Although numerous laboratory studies have also sought to characterize age patterns of risk taking, the degree to which adolescents take more risks than adults varies markedly across studies [3–5].

In recent theoretical work, we have considered how parameterization of laboratory tasks is likely to influence whether or not the expected age pattern in risk taking is observed [4,5]. Specifically, the way in which participants must learn about risk, through description or experience, appears to differentiate tasks in which adolescents take more risks than adults from tasks that less often evince this pattern [5]. To illustrate these two paradigm formats, consider a choice between risky and safe options: 1) an 80% chance of winning $4 but with a 20% chance of winning $0, versus 2) a 100% chance of winning $3. A decision from description (referred to simply as description in this manuscript), would involve merely choosing between these two options, with full knowledge of the probabilities and possible rewards associated with each option. In these types of tasks, at least as typically implemented in experimental studies, adolescents and adults often make similar choices. Conversely, in decisions from experience (referred to as experience), participants make repeated choices from unlabeled options to learn about the probabilities and outcomes associated with each one (e.g., samples from 1) $4, $4, $4, $0, $4…; samples from 2) $3, $3, $3, $3…). Typically, in experience tasks, adolescents take more risks than adults [5].

In the present study, we empirically test how adolescents’ and adults’ choices vary in description compared to experience formats. In this pursuit, we move beyond examining just the overall effects of age on risk taking to gain insights into psychological constructs (e.g., probability weighting, loss aversion, expected-value sensitivity) that may moderate the effects of age on risk preferences across format (description, experience). Another goal of the present study is to explore age differences in decision processes that might yield insights into risk-taking behavior. To this end, we examine age differences in process measures, including eye tracking and pupillometry in description (e.g., does looking more at the risky option correspond to an increased likelihood of choosing that option for both adolescents and adults?) and how participants search for information in experience (e.g., at what point do participants stop sampling and make a choice?).

Perhaps unsurprisingly, our results do not provide straightforward evidence for age differences in decision making. In fact, the findings are in some ways counter to our expectations: Broadly, in our sample, age differences in choice were more pronounced in description than in experience. The absence of significant effects in some analyses may have been due to lack of adequate statistical power, and null results should not be overinterpretted. Nonetheless, we contextualize our findings within a broader literature on adolescent risk taking, and hope that the present work will be useful in helping to leverage ideas from the study of adult decision making toward a deeper understanding of adolescence.

### Psychological constructs that influence risky choice

#### Probability weighting

The notion that there may be different age patterns in description versus experience echoes a finding in the adult judgment and decision making literature—that adults choose differently based on whether risk information is learned through description or experience [6,7]. In description, adults tend to make choices consistent with Prospect Theory (PT) [8,9]. A key component of PT of primary interest in the present investigation is probability weighting, which refers to the idea that probabilities are distorted in decision making. According to PT, people tend to overweight rare (i.e., low-probability) outcomes.

To illustrate the idea of rare-outcome overweighting, we return to the choice problem described above. According to PT, participants will overweight the rare 20% chance of winning nothing, leading them to favor the safe option over the risky option (“risky” is defined here according to the economic definition: the option with higher variability in possible outcomes [10]). While PT is the predominant model describing choices from description, research on experience-based choice demonstrates that adults tend to exhibit the opposite pattern of behavior with respect to probability weighting when they learn about risk through sampling [6]. That is, in experience, where participants draw repeated samples to learn about outcomes from unlabeled options, adults make choices indicating that they underweight rare outcomes [6,10,11]. In experience, relatively lower weighting of the 20% chance of winning nothing would therefore make the risky option more attractive, biasing participants toward taking the risk. This difference in choice biases based on whether information is learned through description or experience is known as the Description-Experience (D-E) gap [6].

Importantly, rare-weighting patterns manifest in differential risk preferences based on the relative favorability of the rare outcome. In the case of the choice problem presented above, the rare outcome ($0 with 20% likelihood) is unfavorable relative to the frequent outcome ($4 with 80% likelihood). In such contexts with unfavorable rare outcomes, rare-overweighting biases decrease the likelihood of risk taking, while the opposite (rare-underweighting) biases increase the likelihood of risk taking. Conversely, in contexts with a rare favorable outcome (e.g., a 90% chance of winning $0 and a 10% chance of winning $32, vs. a 100% chance of winning $3) overweighting the rare $32 outcome would lead to more risk taking, whereas underweighting that outcome would lead to less risk taking. Studies can test for rare-weighting biases by comparing participants’ choices across a set of problems that vary systematically as a function of rare-outcome favorability.

**Fig 1:**
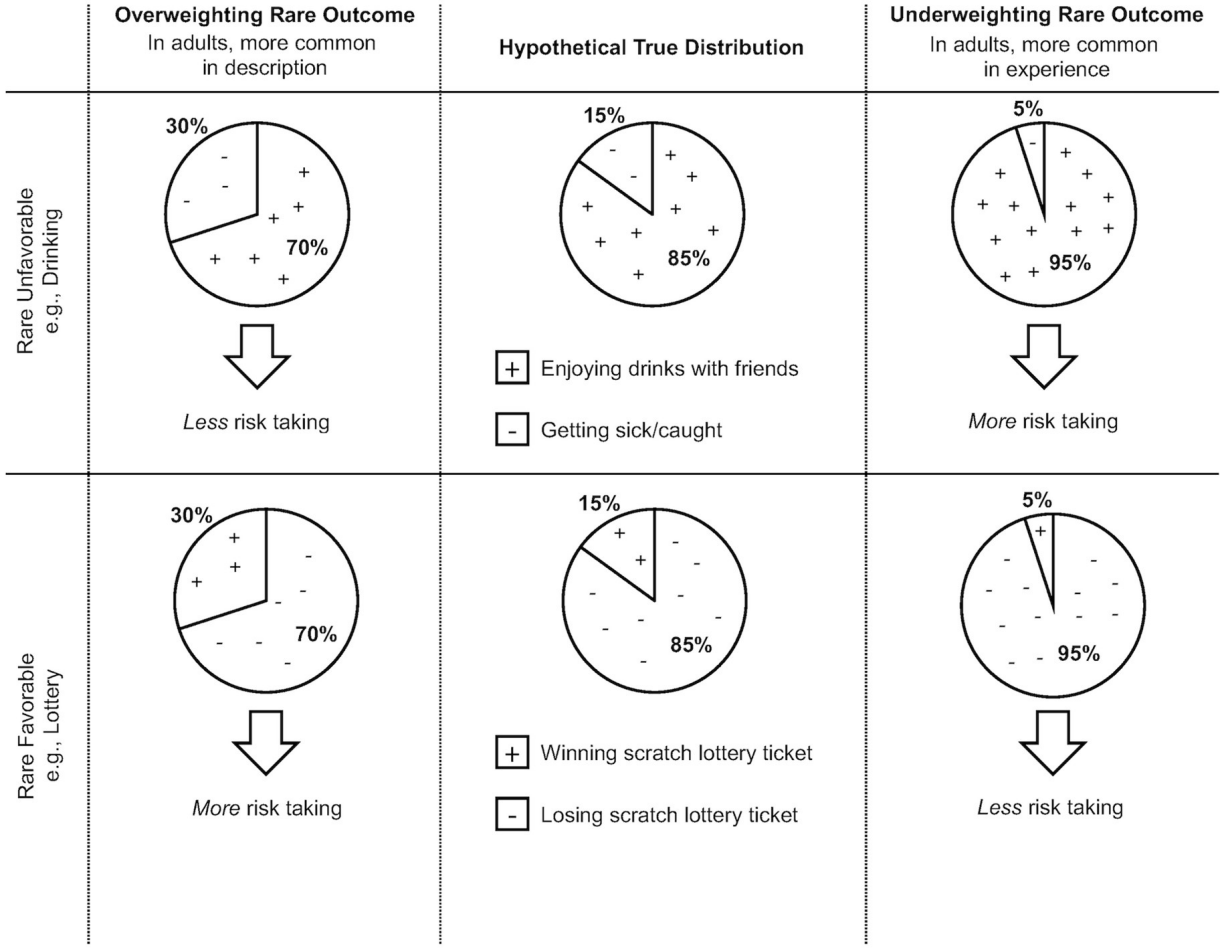
Illustration of rare-weighting biases in relation to choices encountered in the real world.

This framework can be applied to real-world scenarios, including those that are relevant to adolescent behavior. For instance, if an adolescent is deciding whether or not to drink at a party, the possible harmful outcome is typically rare (e.g., the rare and unfavorable outcome of getting caught, Fig 1, top row). In such rare-unfavorable contexts, rare-outcome overweighting would result in a lower likelihood of drinking (Fig 1, top left) while rare-outcome underweighting would lead to an increased likelihood of drinking (Fig 1, top right). On the other hand, individuals may encounter situations in which a rare outcome is favorable, as is the case in buying lottery tickets (a favorable and rare outcome of winning the lottery; Fig 1, bottom row). In such contexts, rare-outcome overweighting would lead to a higher likelihood of buying lottery tickets (Fig 1, bottom left), while rare-outcome underweighting would lead to a lower likelihood of buying tickets (Fig 1, bottom right).

Rare-outcome weighting as a function of decision format has been extensively discussed in the adult literature on the D-E gap (for review, see [7]). In the present study, we explore developmental differences in rare-outcome weighting, a topic that has received little empirical attention, and that may be helpful in understanding age differences in risk taking. Although there is a rich literature on risk taking in adolescents relative to adults, most of the studies in this literature do not include problems with rare outcomes, do not systematically vary the favorability of rare outcomes across problems, or report summarized data that obfuscate age-dependent effects with respect to rare outcomes. However, a handful of prior description studies have presented data such that rare-weighting patterns could be inferred, and show that adolescents’ choices diverged from PT predictions [12–14]. Moreover, some experience-based studies include only options with an unfavorable rare outcome [15–18], and adolescents tend to take more risks than adults in these studies. In such contexts with unfavorable rare outcomes, risk taking may result from relatively enhanced underweighting of rare outcomes in experience.

Additional guidance on possible age differences in choice as a function of task format is provided by a small number of studies directly comparing adolescent to adult decision making in both description and experience. While an early study [19] found that adolescents, like adults, exhibit a D-E gap, elements of the problem set were varied across age groups and the data were not presented in a fashion that permitted contrasts between adolescents and adults. Three other studies, however, provide evidence regarding decision behavior in both description and experience formats in adolescents relative to adults [18,20,21]. In all three studies, relative to adults, adolescents exhibited behavior deemed riskier [18,20] or more tolerant of uncertain outcomes [21]. In contrast, age differences were less pronounced [18,20] or depended on other task-related variables in description-based choices (e.g., different age patterns in gain vs. loss problems; [21]). Although these results are consistent with differential age patterns in rare-outcome weighting within description and experience, no studies have directly examined how rare-weighting patterns differ in the same participants who are asked to make both description- and experience-based decisions.

#### Loss aversion

The present study also offered an opportunity to examine how age differences in choice vary as a function of outcome valence; whether choices involve gains or losses. In description, adults exhibit loss aversion, another component of PT, referring to the idea that losses loom larger than gains that are equivalent in magnitude, and which results in more risk taking in order to avoid a loss than to pursue a win [8,9]. Although loss aversion is frequently observed in adult studies of description-based decision making, it is not typically observed in experience [7]. The available developmental literature that indexes loss aversion in description has demonstrated that adolescents are as loss-averse as adults [21–23]; but see [24,25].

#### Expected-value sensitivity

It is also important to consider whether age patterns in description or experience vary according to the relative advantageousness, or Expected Value (EV), of each option, computed as the sum of each possible outcome value, multiplied by its probability of occurring. It may be the case that adolescents’ heightened risk taking is due in part to difficulty computing which option is most advantageous [26]. Indeed, in many developmental risk taking tasks, it is not possible to disentangle risk taking from EV sensitivity because risky choices are confounded with EV (e.g., the risky option always has a lower EV [27]), so age patterns in risk taking may be partially attributed to differences in EV sensitivity. Prior studies directly investigating adolescents’ ability to choose the option with higher EV are inconsistent [22,25,28–32].

#### Decision processes

Implicit in examining differences in decision behavior as a function of task format and age group is that the processes that support decision making might also change with age. For several decades, studies of adults’ decision making have deployed a variety of process-tracing methods–behavioral or physiological methods that can provide insights into information processing prior to the decision [33]. Very few developmental studies (c.f. [21,34]) employ the process-tracing methods that are commonly used in the adult judgment and decision making literature (for review, see [33]). A present goal was thus to use eye tracking and experience sampling to begin to understand how age differences in processes might relate to age patterns in choice.

#### Eye tracking in decisions from description: Gaze bias and pupil dilation

Eye tracking is frequently used to study decision making in adults [35,36], especially in description studies, but is less often employed to study choice developmentally [37,38]. A large literature demonstrates gaze biases, such that adults look longer at options they ultimately select, and that final fixations prior to making a choice are strongly biased towards the chosen option (e.g., [39–41]). One study demonstrated similar effects in adolescents [34]. Pupil dilation, which can be measured simultaneously with eye gaze position, provides additional insights into physiological processing during decision making, as it is linked with noradrenergic activity in the locus coeruleus (LC; [42]). LC activity, and pupil dilation by proxy, is thought to reflect autonomic arousal, evoked emotion, attention or cognitive load [43–45].

#### Sampling experience: Uncertainty tolerance

In experience, participants learn about options through sampling, and sampling behavior itself can be scrutinized as a process measure in experience [46,47]. For instance, a large number of studies have found that adults rely on relatively few samples when making experience-based choices (e.g., [6]), and one recent developmental study demonstrated that adolescents took fewer samples than adults [21]. The number of samples taken before making a choice is thought to reflect a participant’s attitude towards uncertainty, with less searching reflecting a greater uncertainty tolerance, or a greater willingness to make decisions with less information ([21]; also see S1 File for a related brief discussion and analysis of Sampling Error).

### Study approach and hypotheses

Groups of adolescent and adult participants in the present study were given the same 24 choice problems in both description and experience formats. Problems varied according to the relative favorability of the rare outcome, gain and loss valence, and the relative EV of risky and safe options, allowing us to infer whether psychological constructs related to choice change with age. We also collected eye tracking and experience sampling data to understand how choice processes may relate to age differences in behavior.

A primary aim of the present study was to examine age patterns of decision making using a paradigm and problems similar to those used in adult studies of the D-E gap. We therefore used similar problems and parameters as those in past studies, and accordingly defined rare outcomes as those that make up 20% of the distribution or less [6,7].

Our hypotheses are summarized in Table 1. We expected that, overall, adolescents and adults would take risks at similar rates in description (H1.A.), but that adolescents would take more risks than adults in experience (H1.B.). We also hypothesized that both adolescents and adults would exhibit a D-E gap, and that the D-E gap would be similarly pronounced in adolescents relative to adults (H1.C.).

**Table 1:** Study hypotheses.

Measure	Construct	Hypothesized Age Effect	Observed Age Effect (If Different from Prediction)
1. Risk Taking and D-E Gap	A. Risk taking, Description	Adolescents = Adults	✓
	B. Risk Taking, Experience	Adolescents > Adults	Adolescents = Adults
	C. Description-Experience Gap	Adolescents = Adults	Adolescents < Adults^†^ (still significant in Adolescents)
2: Decisions from Description	A. Overweighting Rare Outcomes	Adolescents < Adults	✓
	B. Loss Aversion	Adolescents = Adults	✓
	C. Expected-Value Sensitivity	Unknown	Adolescents < Adults
	D. Gaze Bias	Adolescents = Adults	✓ (only looking time differed)
	E. Pupil Dilation	Unknown	Adolescents = Adults (more dilation preceding a choice inconsistent with bias)
3: Decisions from Experience	A. Underweighting Rare Outcomes	Adolescents > Adults	✓^†^
	B. Loss Aversion	Unknown	Adolescents = Adults
	C. Expected-Value Sensitivity	Unknown	Adolescents < Adults^†^
	D. Sampling	Adolescents < Adults	✓^†^

^†^ Reflects marginally significant effect.

We further hypothesized that more nuanced differences would emerge when taking into consideration problem-specific contextual features, which can reveal age differences in decision-making constructs and processes. In description, we hypothesized that adults would make choices consistent with overweighting rare outcomes, according to PT predictions, but that adolescents would not (H2.A.). In line with prior research, we expected a similar degree of loss aversion in adolescents and adults (H2.B.). We did not have a strong prediction as to whether EV sensitivity would vary with age in our sample (H2.C.), as past results have been inconsistent in this regard. Process measures in decisions from description were derived from eye-tracking data, and we expected that adolescents would exhibit similar eye gaze patterns to adults (H2.D.). Our pupil dilation analysis was exploratory, so we did not posit any specific directional hypotheses given the dearth of prior research (H2.E.).

We predicted that, in experience, both adolescents and adults would make choices consistent with rare-outcome underweighting, but that this tendency would be exaggerated in adolescents relative to adults (H3.A.). Because loss aversion is not evident in adult experience-based choice [7], we did not expect to find evidence of loss aversion in adults or adolescents (H3.B.). As in description, we did not have a strong hypothesis regarding age differences in EV sensitivity in experience (H3.C.). Finally, in examining experience-sampling process data, we expected that adolescents would take fewer samples than adults, reflecting relatively heightened uncertainty tolerance (H3.D.).

## Materials and methods

### Participants

Participants included a community sample of 31 adolescents (ages 13–17, M = 15.33, SD = 1.30, 21 female) and 33 adults (ages 25–36, M = 29.39, SD = 4.02, 15 female). One adolescent and 3 adults were excluded from all analyses, as they did not respond correctly to any comprehension questions about task instructions (listed in S1 File), leaving a final sample with 30 adolescents and 30 adults. This sample size is aligned with prior studies comparing adolescent to adult risky decision making. In a recent meta-analysis where aggregated results showed higher laboratory risk taking in adolescents versus adults [3], individual studies included a median of 27 adolescents and 26 adults.

### Behavioral tasks

#### Decision-making task

**Fig 2:**
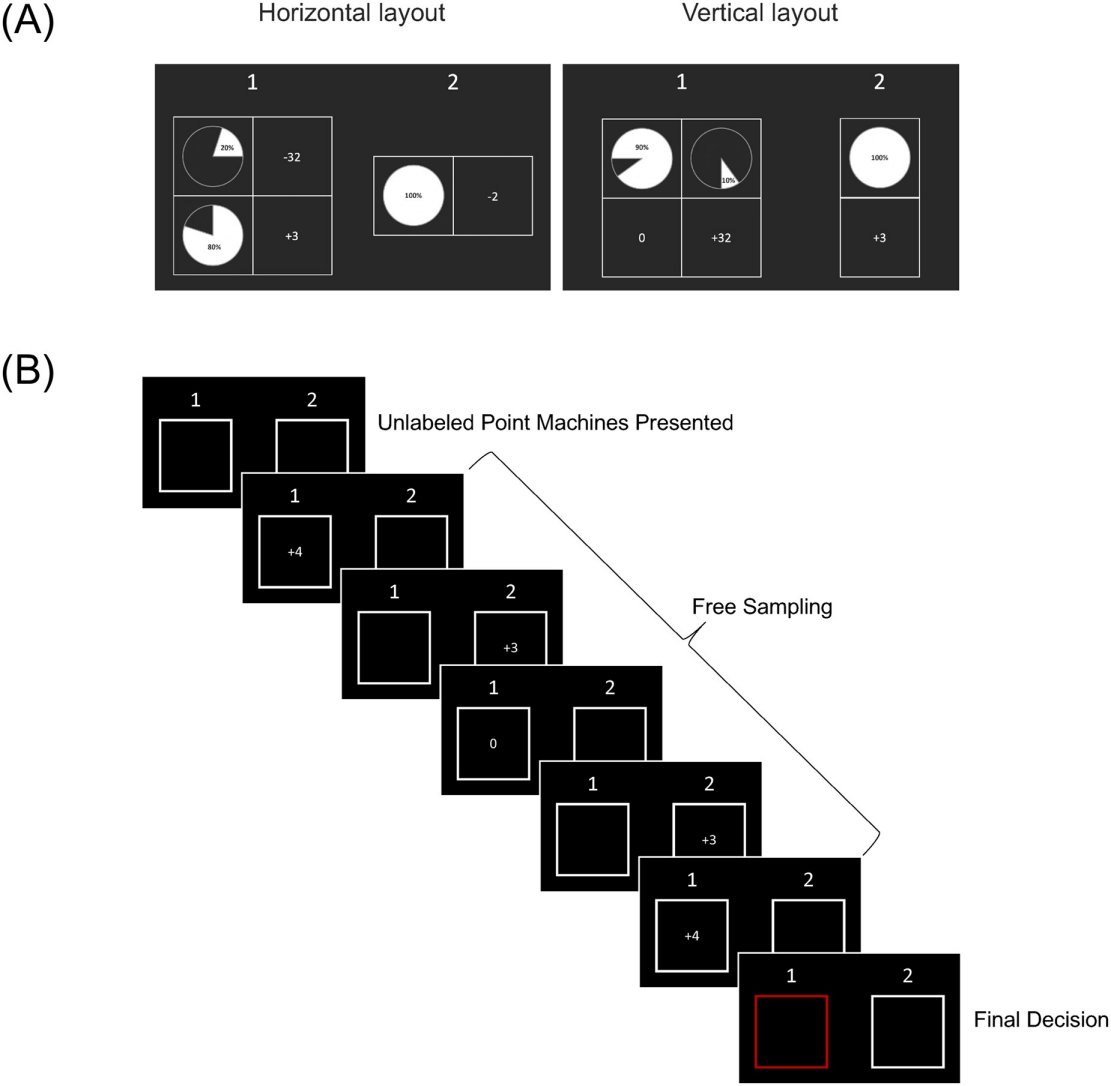
Task schematic. (A) Decisions from description layouts displaying two representative options. The layout type (horizontal [left] or vertical [right]) was randomized on every trial. The side of the screen on which the risky and safe options appeared, and the position of the rare and frequent outcomes within the risky grid (top/bottom in horizontal layout, left/right in vertical layout) were also randomized. (B) A representative trial from the decisions from experience format. Participants were presented with unlabeled point machines and sampled from the machines as many times as they wanted. After each sample, an outcome from that machine was drawn based on the underlying probability distribution and shown for 1s. Participants pressed enter to indicate they were ready to make their final decision and then indicated their choice on the final decision screen.

Age differences in choice were assessed using a within-subjects paradigm, where participants encountered the same choices in both description and experience formats. Participants were presented with a series of problems requiring a choice between two alternative “point machines.” In counterbalanced blocks, decisions were based on explicit descriptive information regarding the values and outcome probabilities assigned to each machine (Fig 2A), or on experience obtained by sampling outcomes from those machines in the absence of explicit information about the machine values or probabilities (Fig 2B). In description, probabilities were presented as pie charts with overlaid numeric percentages. Each of the problems used in the task was presented in both formats, in pseudo-random order. Participants completed a cognitively demanding filler task (Go/No-Go Continuous Performance Task) between description and experience blocks to limit the likelihood of any carryover effects from problems encountered in the first-round format. There was no effect of task order on the proportion of choices consistent with PT predictions in description or experience (see S1 File).

*Decision problems*. In the present study, problems always included one risky option (a machine that produces a likely outcome paired with a rare outcome) and one safe option (a machine with one fixed outcome), as this type of problem is most likely to evince a D-E gap [7]. The 24 problems are listed in S1 Table in S1 File. Four of the problems (1, 7, 13, and 19) originated from a seminal study on the D-E gap [6], and have since been used in many D-E gap studies [7]. These four problems are also listed as the example problems in Table 2. Our larger, more variable, problem set preserved the core dimensions of the original problems, and varied according to rare-outcome favorability (favorable rare outcome or unfavorable rare outcome) and valence (gain or loss; [48]). This resulted in 4 problem types: A) Rare Unfavorable, Loss; B) Rare Unfavorable, Gain; C) Rare Favorable, Loss; D) Rare Favorable, Gain. Further, while most D-E gap studies present single-valence problems, where all outcomes are either gains or losses but not both [7], our problem set included two blocks of problems presented in counterbalanced order: one block with single-valence problems, and another block with mixed-valence problems where the risky option yielded both a gain and a loss outcome (although the valence of the risky option’s expected value, or EV, was always consistent with the valence of the safe outcome). Finally, we varied the relative EV of risky and safe options: In some problems, the EV of the risky option was higher than the safe option, in some problems the opposite was true, and in some problems risky and safe options had the same EV.

**Table 2:** Choice data collapsed by problem type.

Problem Type	Rare Favorability	Valence	PT-Predicted Option	Example Risky Option	Example Safe Option	Adolescents			Adults		
						Desc. % Choosing PT Option	Exp. % Choosing PT Option	D-E Gap	Desc. % Choosing PT Option	Exp. % Choosing PT Option	D-E Gap
A	Unfavorable	Loss	Safe	90%: 0, 10%: -32	100%: -3	54	42	12*	61	43	17**
B	Unfavorable	Gain	Safe	80%: +4, 20%: 0	100%: +3	56	37	19***	69	39	30***
C	Favorable	Loss	Risky	80%: -4, 20%: 0	100%: -3	47	33	14*	60	40	20***
D	Favorable	Gain	Risky	90%: 0, 10%, +32	100%: +3	38	35	3	51	40	11†

PT-Predicted option lists the favored option, risky or safe, based on Prospect Theory predictions. Example risky and safe options represent a sample problem within this problem type. These problems were also presented in [6,49]. Asterisks in the D-E gap columns represent the significance of a chi-square test of the D-E gap collapsed choice data within an age group. PT = Prospect Theory. Desc. = Description. Exp. = Experience. D-E Gap = Description-Experience Gap.

^†^p < .1,

*p < .05,

**p < .01,

***p < .001.

#### Probability estimation

As a validity check on participants’ ability to form outcome probability estimates from experience sampling, participants completed a probability estimation task. On each trial, participants were presented with two unmarked bins, each containing shapes (circles, stars, triangles, diamonds), and sampled freely until they felt confident reporting an estimate of the contents of each bin. Finally, participants used a slider bar to report their estimates for the distribution of shapes in each bin. The shape probability distributions paralleled those in the experience point machines: one of the bins only contained one shape (analogous to safe point machines) and one bin contained a rare shape and a frequent shape (analogous to risky point machines). Participants completed 4 trials of this task: 2 where the rare shape comprised 20% of the distribution and 2 with a rare shape that comprised 10% of the distribution, in random order.

### Eye tracking setup

To better understand decision processes in description, we measured eye gaze and pupil size using a Tobii T60XL eye-tracking system at 60hz. The environment (lighting, sound) was controlled across participants, and stimulus positioning (the position of the risky vs. safe option on the left vs. right; the position of the point values and probabilities on the left vs. right and top vs. bottom) was randomized to account for biases in gaze patterns.

### Procedure

After obtaining written informed consent from adult participants, and written parental consent and child assent from adolescents, decisions from description and experience were administered in a counterbalanced fashion, with the Go/No-Go filler task separating choice formats. The probability estimation task always followed both decision formats. Finally, participants were asked a series of questions about the instructions (see S1 File) to ensure understanding. All procedures were approved by the Temple University Institutional Review Board.

### Data analysis

Analyses were run in R version 3.5.1 [50]. We assessed the D-E gap by computing chi-square tests on choices in description versus experience, as a function of problem type and age group (Table 2). For a fine-grained analysis of choice data, we ran a series of generalized linear mixed-effects logistic regressions (glmer; lme4 package; [51]), using choice context (rare favorability, problem valence, EV) to predict risk taking. All models included problem features as fixed effects with random intercepts for each subject. Models used the BOBYQA optimizer, and estimates were computed with Gauss-Hermite approximation of the likelihood with 10 integration points. We plotted all significant and marginally significant interactions for interpretation using sjPlot [52]. Experience regressions included only trials where participants experienced all possible outcomes (the safe outcome from the safe machine and both the frequent and rare outcomes from the risky machine); 62% of trials on average. On those trials, both rare and frequent outcomes were included in the analyses. The percent of trials included did not differ significantly by age group (see Sampling Error section in S1 File). Effect sizes were very similar when regressions included all trials.

In analyses of sampling and probability estimation, along with RT and sampling bias (S1 in S1 File), we compared group means between adolescents and adults. Before running these tests, we removed outliers (greater than 1.5 times the interquartile range) within each individual’s choices and took the mean of the remaining values for each individual. Then, we removed any participants who were outliers for that measure within their age group for each analysis. For the D-E gap analysis, one adult was removed. For the sampling analysis, 3 adolescents were removed. In the probability estimation task, there were 2 adults removed from the analysis of age differences in sampling, and 3 adolescents removed from the analysis of age differences in sampling bias. In the analysis of overall risk taking, 3 adolescents and one adult were removed. We ran t-tests to compare age groups if the data met test assumptions. Otherwise, we ran non-parametric Wilcoxon rank-sum tests.

#### Eye tracking in description

Eye tracking data could not be collected for one adolescent, who needed to wear thick glasses throughout the experiment, and for one adult due to experimenter error. Data were preprocessed using in-house scripts in MATLAB (see S1 File) and then transferred to R for use as predictors in glmer models as described above. For 4 adolescents and 1 adult, fewer than 50% of eye tracking acquisitions were valid, so their eye-tracking data were excluded from all analyses. The final sample for eye-tracking analyses was therefore 25 adolescents and 28 adults. Within that final sample, there was not a significant age difference in the number of valid eye tracking acquisitions (Mdn_adolescents_ = 87%; Mdn_adults_ = 90%; W = 263, p = .124). Because we excluded several participants and individual trials in these eye tracking regressions that did not need to be excluded in the behavioral glmer regressions, we reran description behavioral regressions in the subset of participants and trials with valid eye tracking data. All behavioral effects from the full sample were significant within this subsample.

*Computing change in pupil dilation*. For each description problem for each participant, we first computed a baseline pupil diameter from the 100ms preceding stimulus presentation. Next, we measured the percent change from baseline diameter in the last 1/3 of the trial, the time period directly before the participant made a choice, and used this variable as a predictor in glmer models. In all regressions with pupil data, we included proportion of looking time in the brightest areas of interest (risky frequent and safe pie charts, which were mostly white in contrast to the black background) as a covariate, as looking more often at these areas would also increase pupil constriction due to increased light exposure.

## Results

### Risk taking and D-E gap

For comparability with prior developmental work comparing risk taking in adolescents and adults (e.g., studies reported in the Defoe et al., 2016 meta-analysis), we began by running a repeated-measures 2 (format: description, experience) x 2 (age group: adolescent, adult) ANOVA on risky choices. Interestingly, there were no significant main or interactive effects of age group on risk taking (see S1 File for full results, Risk Taking in Description and Experience section). In other words, based on this more traditional approach we found no overall age differences in mean risk taking in description (aligned with H.1.A.), or experience (counter to H.1.B.).

Next, we investigated whether participants exhibited a D-E gap, making choices consistent with rare-outcome overweighting in description but rare-outcome underweighting in experience. Table 2 displays choices for each “problem type” for each age group. In problems with rare outcomes that are unfavorable (types A and B), Prospect Theory (PT) predicts that participants will be more likely to choose the safe than the risky option. Conversely, for problems with favorable rare outcomes (types C and D), choosing the risky option over the safe option would be consistent with PT predictions. Based on the prior D-E gap literature, the proportion of participants choosing the PT-consistent option in description should be greater than the proportion of participants choosing that option in experience [6]. We computed the D-E gap as: Description % Choosing PT Option—Experience % Choosing PT Option. We found a significant D-E gap in the predicted direction for each problem type in each age group (Table 2), except with the rare favorable gain problems (type D) where the gap did not reach significance for either adolescents or adults.

We subsequently assessed age group differences in the D-E gap. To this end, we ran a 2 (format: description, experience) by 2 (age group: adolescent, adult) repeated-measures ANOVA on the proportion of choices consistent with PT predictions. We found a marginally significant format x age group interaction (F(1,112) = 2.77, p = .099, η^2^_p_ = .02). Follow-up t-tests suggested that the proportion of PT-consistent choices was significantly higher in description compared to experience for both adolescents (M_description_ = .49, M_experience_ = .37; t(29) = 3.66, p < .001, r = .56) and adults (M_description_ = .61, M_experience_ = .39; t(28) = 5.53, p < .001, r = .72). In additional follow-up tests, we found that the proportion of PT-consistent choices did not significantly vary between adolescents and adults in experience (t(56.92) = .45, p = .66, r = .06). However, in description, adolescents made significantly fewer PT-consistent choices than adults (t(56.80) = 2.32, p = .02, r = .29). Importantly, this variability in choices across age groups, and within age groups across problem types, encouraged finer-grained trial-level analyses of the choice data, as well as process data, as presented below.

As a preliminary analysis, we also ran linear regressions to test whether risk taking or the proportion of PT-consistent choices in either description or experience varied with linear age within our adolescent sample. None of these four regressions reached significance (ps >.11; see S1 File for full results, Testing for Linear Age Effects section).

### Decisions from description

To gain further insight into age patterns in choice behavior during description, we ran several glmer models, which allowed us to test whether choices systematically differed by age group and/or as a function of contextual variables, while controlling for subject-level differences. In every regression, the outcome variable was whether or not the participant chose the risky option (risky choice was coded as 1 and safe choice was coded as 0). For each predictor, we estimated an Odds Ratio (OR), which can be interpreted as the odds of risk taking for the higher coded level of each predictor variable (or for the continuous variable, for larger values of the predictor). Rare Favorability was coded as -.5 when the rare outcome was unfavorable and.5 when the rare outcome was favorable. Valence was -.5 for loss problems and.5 for gain problems. Expected Value (EV) difference was entered as a continuous predictor, computed as the difference in the EV of the risky option relative to the safe option. We started with a base model that included each of these predictors, along with age group (-.5 = adolescents,.5 = adults).

**Fig 3:**
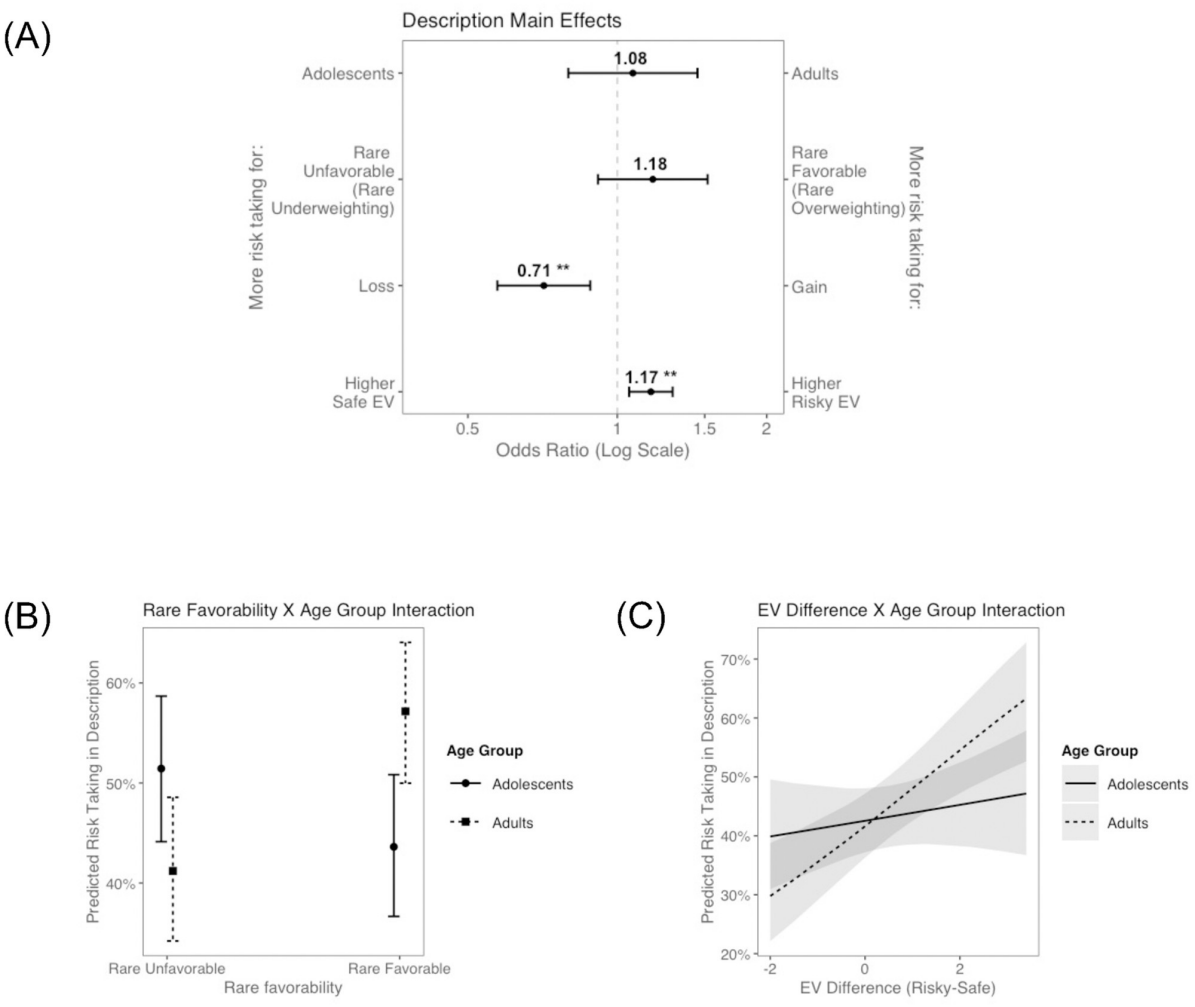
Results from decisions from description regressions. (A) Main effects in the base model. (B) Significant interaction effect–Rare Favorability X Age Group. (C) Significant interaction effect–Expected Value Difference (Risky-Safe) X Age Group. **p < .01.

Results are displayed in Fig 3a. As expected, participants took more risks when the risky option had a higher EV than the safe option (b = 0.16, OR = 1.17, p = .002). Participants also took more risks in loss problems relative to gain problems (b = -0.34, OR = 0.71, p = .002), evidence for loss aversion [8]. Interestingly, there was no main effect of rare-outcome favorability, suggesting that in the full sample (mixing adolescents and adults) there was not a bias towards over or underweighting rare outcomes (b = 0.16, OR = 1.18, p = .204). As observed in the ANOVA analysis, there was no main effect of age group on risk taking in description (b = 0.07, OR = 1.08, p = .636).

We next tested whether each problem-type predictor interacted with age group to predict risk taking. There was a significant interaction between rare-outcome favorability and age group (Fig 3b; b = 0.96, OR = 2.61, p < .001), such that only adults’ choices were consistent with the weighting pattern predicted by PT (Table 1, H2.A.). That is, adults took more risks when the rare outcome was favorable than unfavorable, as if they were overweighting rare outcomes. In contrast, adolescents’ choices were more consistent with underweighting of rare outcomes in description. There was no significant interaction between valence and age group (H2.B; b = -0.14, OR = 0.87, p = .518), pointing to similar loss aversion in adolescents as in adults. Additionally, the exploratory EV by age group interaction (H2.C.) reached significance, such that adults were more sensitive to EV differences than adolescents (Fig 3c; b = 0.20, OR = 1.23, p = .018).

To ensure that we were not missing a more subtle age effect in description choices, we tested whether log-transformed response time (RT) varied by age group, and whether age group interacted with problem-level variables to predict RT in lmer regressions. None of these analyses yielded significant age differences in RT (ps >.45; see S1 File for full results, Description Response Time section).

We used eye tracking to examine whether gaze biases or pupil dilation might explain differential choice patterns in description. Consistent with past literature [39–41], we found a significant recency gaze bias, such that the last item fixated prior to choice was also more likely to be chosen (glmer model predicting risky choice with a binary variable coded for whether the risky option was last fixated, b = 2.98, OR = 19.68, p < .001). There was no interaction between age group and last item fixated (b = 0.01, OR = 1.01, p = .982), suggesting that the recency effect was similarly strong in adolescents and adults.

**Fig 4:**
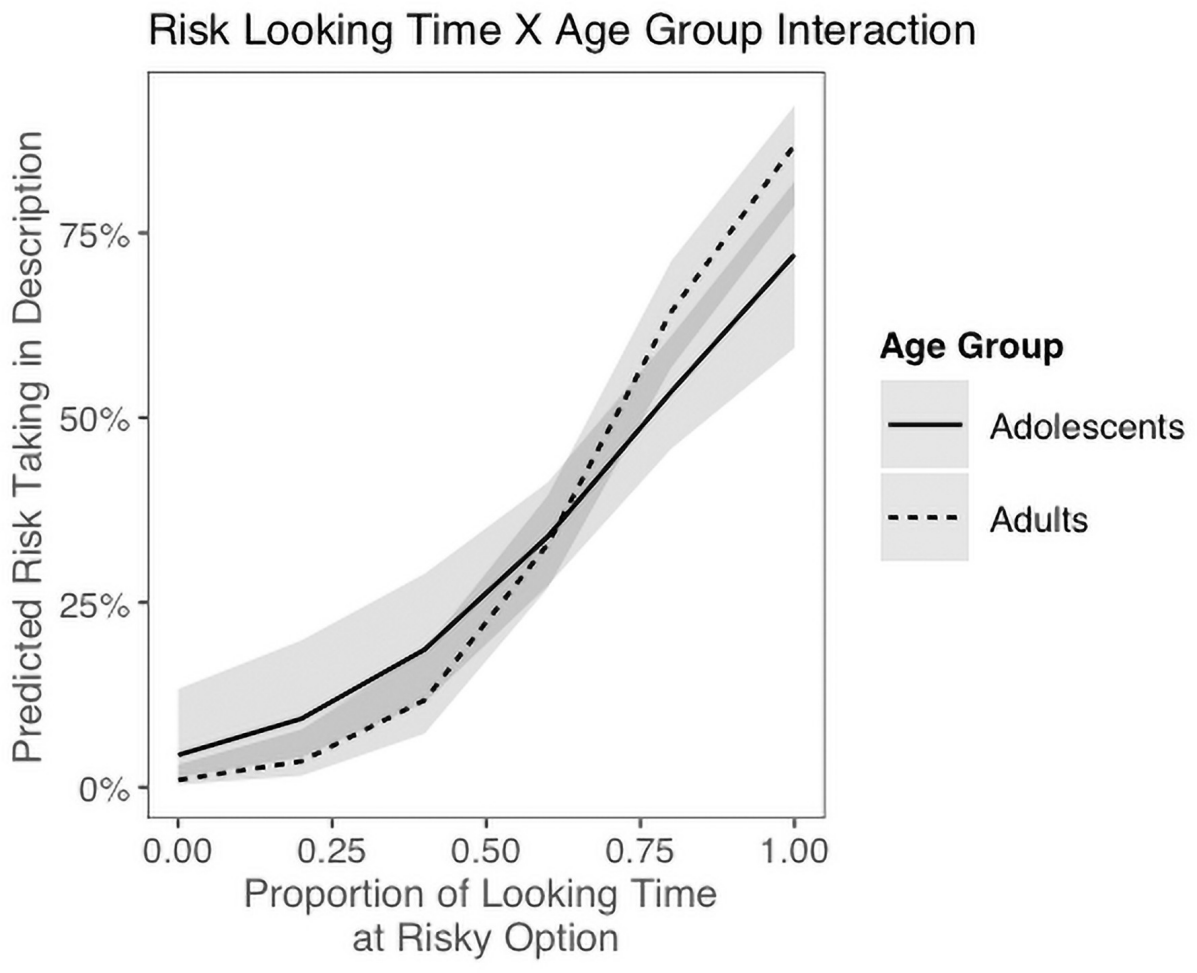
Looking time by age group interaction. In decisions from description, a significant interaction between the proportion of looking time at the risky option and age group to predict the likelihood of choosing the risky option. Adults were more likely than adolescents to choose the option they looked at longer.

We also examined whether participants looked more at the options they ultimately chose, and whether this effect differed with age. We indeed found evidence for a gaze bias, with a larger proportion of gaze time at the risky option predicting risky choice (glmer, gaze time main effect: b = 5.31, OR = 202.30, p < .001). Interestingly, there was a significant interaction between looking time and age group, where adults’ looking time at the risky option was more predictive of choice than adolescents’ (b = 2.47, OR = 11.82, p = .040; Fig 4). Together, eye gaze results suggest that gaze biases are similar in adolescents and adults, but may be slightly more pronounced in adults (H2.D.).

**Fig 5:**
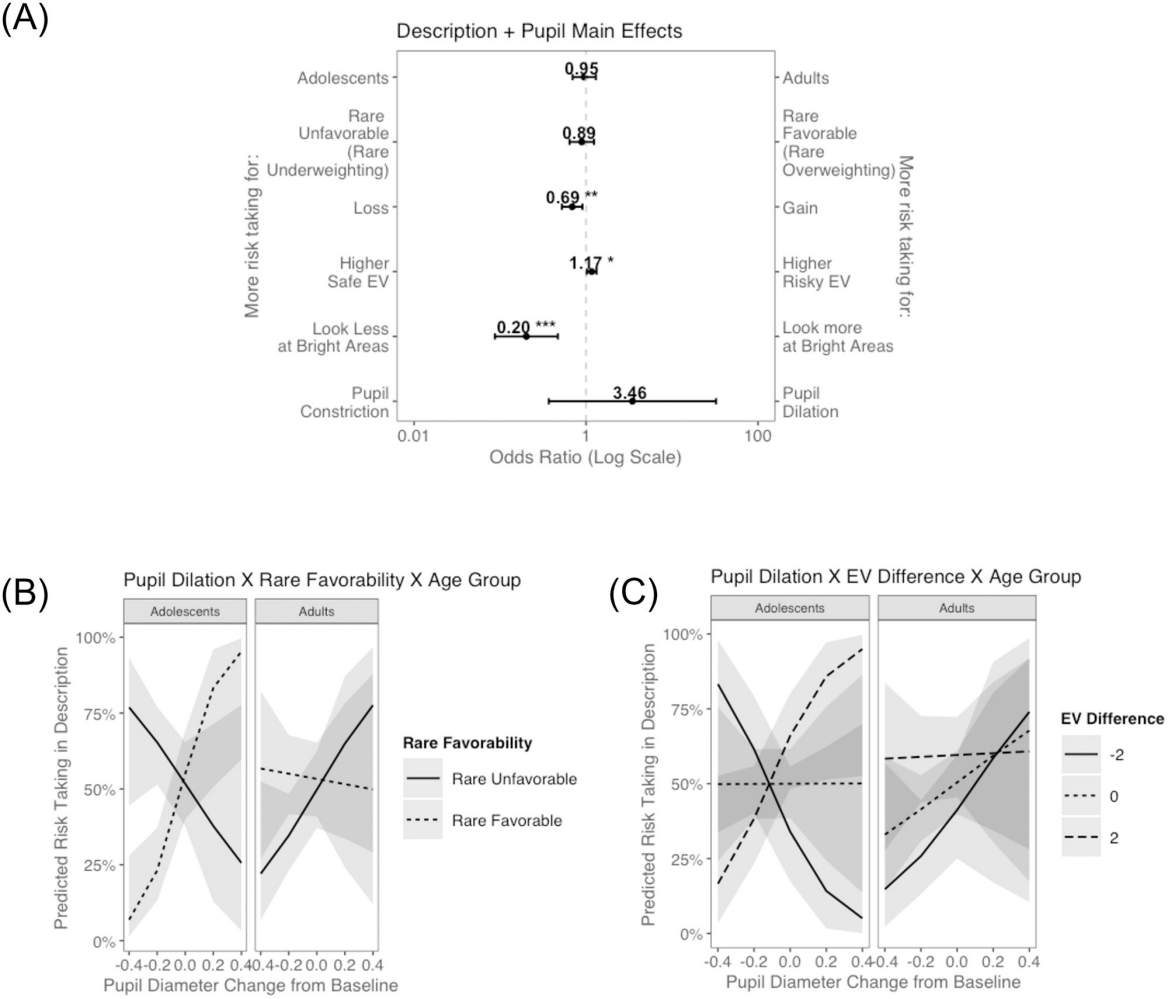
Results from description models with pupil dilation data. Main and interaction effects from description glmer models that included pupil dilation (indexed as proportion change from baseline). (A) Main effects from the base model. (B) Significant interaction effect–Rare Favorability X Age Group X Pupil Dilation; (C) Significant interaction effect–Expected Value Difference (Risky-Safe Option) X Age Group X Pupil Dilation. *p < .05, **p < .01, ***p < .001.

Next, we examined whether pupil dilation related to risk taking in description differentially as a function of age (H2.E.). We wondered whether whether pupil dilation in the moments preceding choice interacted significantly with age group and behaviorally significant problem-level variables (rare outcome favorability or EV; Fig 5a).

There was a significant three-way interaction between rare favorability, age group and pupil dilation (Fig 5b, b = -13.33, OR = 0.00, p = .003): in adolescents, higher pupil dilation was predictive of more risk taking when the rare outcome was favorable, whereas the opposite pattern was found when the rare outcome was unfavorable. Recall that adolescents took more risks when the rare outcome was unfavorable than when it was favorable. It seems that more dilation coincided with choices opposite to this decision strategy (e.g., when they decide to take a risk on a rare-favorable problem). This pattern also held true in the adult sample, although the effect was not as strong (i.e., the slopes of each favorability condition appear to be more similar). Because adults exhibited the opposite behavioral pattern from adolescents, the opposite-sloping lines in Fig 5b. indicate that adults’ pupils dilated more for choices opposite to their decision bias (i.e., higher pupil dilation for a decision to take a risk when a rare outcome is unfavorable).

The interaction between EV difference, age group and pupil dilation was also significant (Fig 5c, b = -3.68, OR = 0.03, p = .033). In adults, less pupil dilation was associated with less risk taking, especially when EV was much lower in the risky than the safe option. Adolescents took more risks when pupil change was higher and the EV difference was large (regardless of which option had a higher EV). In sum, pupil dilation patterns appear to reflect age differences in decision biases.

### Decisions from experience

To understand choices in the experience format, we ran a series of glmer models similar to those in description. In order to control for error in sampling (i.e., that the sampled outcomes differed for every participant), we computed the EV of each option based on the participant’s sampling experience, rather than on the true underlying distribution. As a hypothetical example, if a participant sampled the outcomes 4, 4, 0, 4, 4, 4, the participant’s experienced EV for the risky option would be (4+4+0+4+4+4)/6 = 3.33. If every sample for the safe option yields 3 points (with a corresponding EV of 3, as in choice problem 7), the experienced EV difference variable for this problem and this participant would be 3.33–3 = 0.33.

**Fig 6:**
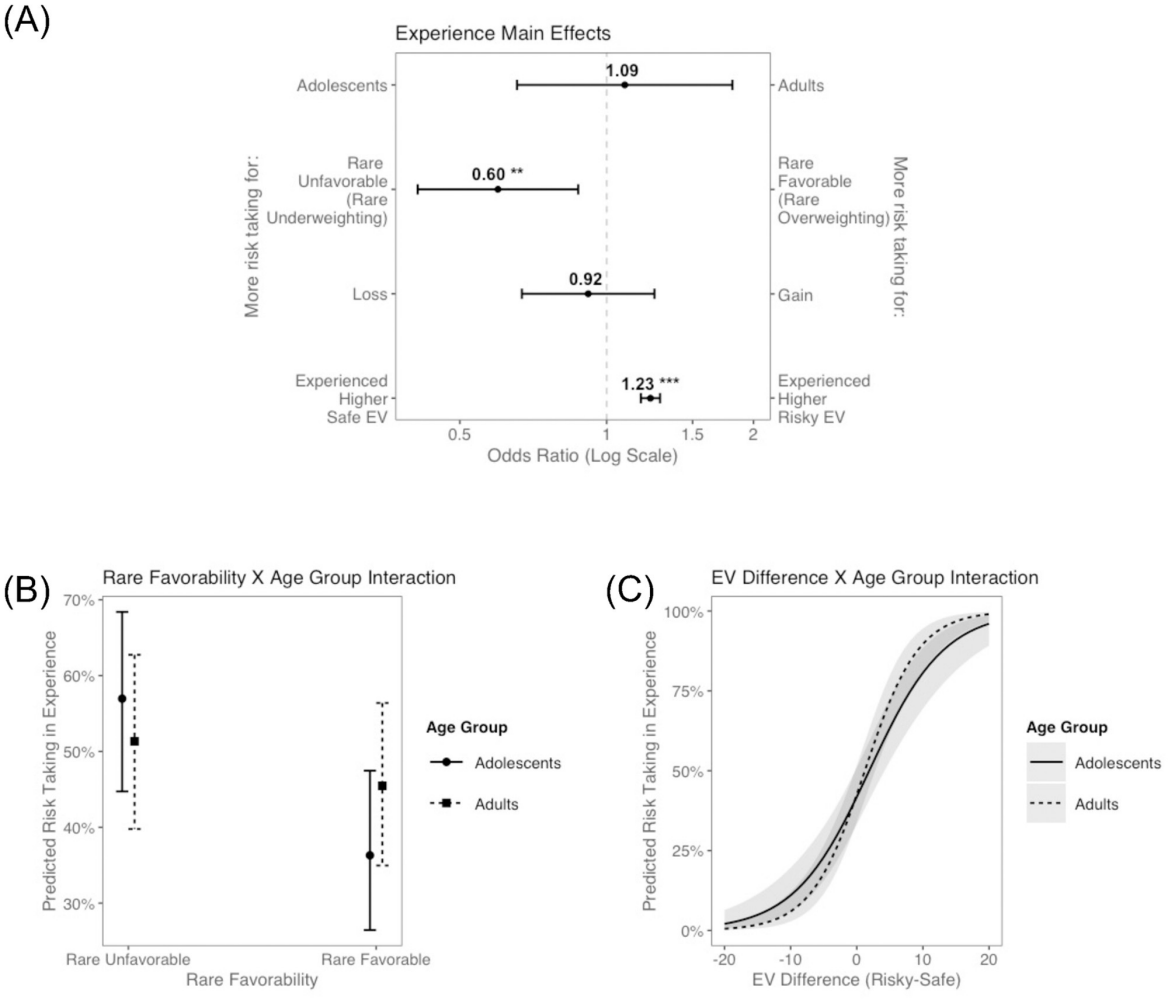
Results from glmer models in decisions from experience. (A) Main effects in a base model. (B) Marginally significant interaction effect–Rare Favorability X Age Group. (C) Marginally significant interaction effect–Experienced Expected Value Difference (Risky-Safe) X Age Group. **p < .01, ***p < .001.

Results are displayed in Fig 6. As expected, participants took more risks when the EV of the risky option was highest (b = 0.21, OR = 1.23, p < .001). Participants also took more risks when the rare outcome was unfavorable (b = -0.51, OR = 0.60, p = .007), in line with the broader D-E gap literature, where participants are likely to underweight rare outcomes in experience. Risk taking did not differ as a function of valence (b = -0.09, OR = 0.92, p = .584) or age group (b = 0.09, OR = 1.09, p = .742).

In our analysis of problem-level contextual variables, we found a marginally significant interaction between rare-outcome favorability and age group (b = 0.61, OR = 1.84 p = .052; Fig 6b), consistent with the hypothesis that adolescents exhibit more pronounced rare-outcome underweighting (H3.A.). Furthermore, adults appear to be marginally more sensitive to experienced EV than adolescents (b = 0.07, OR = 1.07, p = .055; Fig 6c; H3.C). The interaction between valence and age group was not significant (b = 0.24, OR = 1.27, p = .448; H3.B.).

We next turned to an analysis of sampling patterns in experience to understand how information seeking might change with age. In line with our expectations (H3.D.), adults sampled more than adolescents marginally overall (Mdn_adolescents_ = 10.35, Mdn_adults_ = 12.74, W = 282.50, p = .051, r = .18) regardless of whether the option was risky (Mdn_adolescents_ = 5.27, Mdn_adults_ = 6.32, W = 277.50, p = .027, r = .29) or safe (Mdn_adolescents_ = 4.92, Mdn_adults_ = 6.13, W = 285.00, p = .036, r = .28).

### Probability estimation from experiential learning

To gain further insight into age differences in sampling behavior and the ability to form accurate internal probability representations from sampling, participants completed an additional probability estimation task. We explored whether the marginal age differences in experience choice patterns might be due to age differences in the ability to estimate probabilities based on sampling. Participants sampled from unlabeled distributions of shapes and were asked to estimate the proportional distribution of each shape. We computed the degree to which the estimation of the rare shape deviated from the participant’s actually experienced distribution of that shape. Importantly, there was virtually no difference between adolescents’ and adults’ estimation error (Mdn_adolescents_ = .04, Mdn_adults_ = .03, W = 398.50, p = .92, r = .01), suggesting that adolescents are able to estimate outcomes from sampled distributions similarly to adults. Interestingly, in this task, adolescents sampled more than adults (M_adolescents_ = 29.32, M_adults_ = 22.85, t(56) = 2.17, p = .034, r = .28), potentially indicating a lower tolerence for uncertainty when asked to attend to features of the probability distribution.

## Discussion

In the present study, we examined risky decision making in a sample of adolescents and adults. Participants made the same 24 choices from both description and sampling experience. Decision problems varied systematically on contextual factors, including favorability of the rare outcome, valence (gain or loss), and expected value (EV) of the risky and safe options. This design allowed us to test how psychological constructs related to choice influence decision making as a function of age group. We also explored possible age differences in decision processing as indexed by eye-tracking measures in description, and by experience sampling behavior.

Although we found several age differences in decision making, which will be discussed in further detail below, many features of adolescent and adult decision making were similar, or at least effects of age group were not sufficiently large to be detected with the current sample size. Adolescents and adults took risks at overall similar rates in both description and experience, and the D-E gap was significant in both age groups. In description, both adolescents and adults took more risks in loss than gain problems, consistent with the idea that participants exhibited loss aversion [9], and the degree of loss aversion did not differ by age group. Further, several of our process measures yielded similar results across age groups. In description, both adolescents and adults looked last at the option they ultimately chose, and there was no difference in description response time across age groups. In experience, although adolescents sampled less frequently than adults, the effect was small and did not result in a significant difference in sampling error. These process-level similarities suggest that adolescents in our study made decisions that were as careful and informed as those of adults.

**Fig 7:**
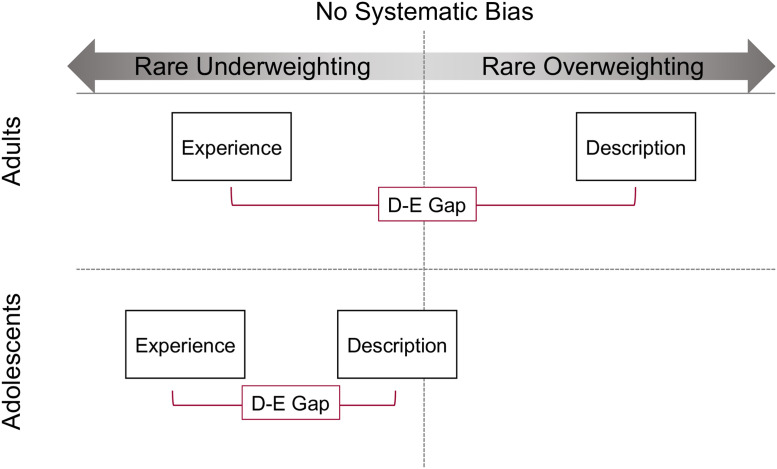
Graphical depiction of age patterns in rare-outcome weighting. Adults (top) make choices as if they overweight rare outcomes in description (consistent with Prospect Theory predictions) and underweight rare outcomes in experience. Adolescents appear to underweight rare outcomes in both description and experience, but this underweighting is more pronounced in experience.

However, in further analyses that accounted for variation in choice as a function of problem-level contextual features, we found that adolescents’ and adults’ choices varied in several respects. Whereas adults made choices as if they overweighted rare outcomes in description-based decision making, which is consistent with predictions based on prospect theory (PT; [8]), adolescents did not exhibit this bias. In marked contrast, they appeared to underweight rare outcomes in description (Fig 7). This age-related difference in choice was also reflected in pupil dilation, which was greater immediately prior to choices that were counter to participants’ choice biases.

In experience, age differences in choice with respect to rare-outcome weighting were more subtle. Both adolescents’ and adults’ choices were consistent with underweighting of rare outcomes, but there was some evidence to support our hypothesis that adolescents had a more pronounced rare-underweighting bias than adults after controlling for individual differences in sampling experience.

These results may also be understood by underscoring the relative consistency of adolescents’ choice behavior in description and experience formats. Although the phenomenon was much more pronounced in experience than description, adolescents appeared to underweight rare outcomes in both contexts (Fig 7). In contrast, adults’ behavior diverged more based on choice context: adults chose as if they overweighted rare outcomes in description but underweighted them in experience. Thus, the D-E gap observed in adults is explained by a shift from overweighting of rare outcomes in description to underweighting of those outcomes in experience, while the D-E gap observed in adolescents is driven only by a more exaggerated underweighting of rare outcomes in experience relative to description.

We also found that adolescents were less sensitive to expected value (EV) than adults in description and marginally in experience. In other words, adolescents were less likely than adults to choose the mathematically advantageous option. However, among adolescents, advantageous choices coincided with greater pupil dilation, perhaps reflecting greater sensitivity to anticipated rewards, or, alternatively, reflecting increased attention or difficulty prior to making advantageous choices. Pupil dilation patterns with respect to EV were slightly different among adults, with greater pupil dilation associated with riskier choices. This effect was especially pronounced when the risky option had a lower EV, possibly reflecting heightened arousal before adults took a disadvantageous risk.

Our study presents a new approach to investigating the roots of adolescent risk taking by examining choices through the lens of the judgment and decision-making literature. In the majority of empirical studies, adolescents take more risks than adults [3], especially in experience-based tasks [5]. This age pattern in risk taking emerges even in studies with similar sample sizes to our study (e.g., those discussed in [3]), though we did not find evidence for this pattern in the present data. Rather, in the present study, risk taking depended on multiple problem-specific contextual factors that have been more comprehensively considered in the adult decision-making literature than in the developmental literature, and that reflect likely age differences in psychological constructs that drive choice. Of particular interest are our description results with regard to PT, which includes indices of two major psychological constructs: loss aversion and probability weighting. Within description, prior research has found that adolescents and adults are similarly loss-averse [21–23], and we replicate this finding. However, our results indicate that probability weighting in adolescents may not be aligned with PT predictions, as adolescents in our study slightly underweighted, rather than overweighted, rare outcomes from description. Although ours is the first developmental study to directly examine probability weighting by systematically varying rare-outcome favorability, a few prior studies presented data such that rare-weighting patterns could be inferred, and similarly found that adolescents’ choices diverged from PT predictions [12–14]. Our pupil dilation findings lend further support to the idea that adolescents and adults might make different decisions with respect to rare outcome favorability. Collectively, these results suggest that loss aversion and probability weighting biases may develop independently of one another. Future work would benefit from directly charting the progression of PT biases from adolescence through adulthood.

Eye tracking is not often utilized in developmental decision making studies [37], and this study demonstrated its utility for understanding choice processes in concert with behavioral data. Our eye-gaze data are well-aligned with the only prior study using eye tracking to index decision making processes in adolescents and adults during description [34]. Specifically, we found that like adults [39–41], adolescents exhibit gaze biases such that they look at the chosen option immediately prior to choice. Furthermore, both adolescents and adults look longer at the option they ultimately choose, but this effect is less pronounced in adolescents than adults. This result suggests that there may be age differences in the dynamics of information integration during decision making.

The present study also makes a unique contribution by examining pupil dilation, an index of autonomic arousal [45], to aid in interpreting choice data. Both adolescents and adults appeared to exhibit weighting biases in decision making (rare-outcome overweighting in adults and underweighting in adolescents), and more pupil dilation coincided with choices opposite to that bias. Past work has shown distinct neural signatures when choices are opposite to an individual’s decision biases [53], and such choices also evoke increased arousal and effort, along with pupil dilation [54]. In sum, the differential choice patterns in adolescents and adults, paired with different arousal and effort patterns reflected in pupil dilation, lend further credence to the notion that PT biases are still developing in adolescents.

We also extended work on experiential decision-making paradigms in the developmental literature. The majority of developmental experience studies have involved some variant of the Iowa Gambling Task (IGT) [55], which requires making sequential choices between two advantageous and two disadvantageous decks of cards. These developmental IGT studies typically show more choices from the riskiest disadvantageous deck in adolescents relative to adults [15–18]; but see [56]. However, in the IGT, the riskiest deck, which yields frequent high rewards paired with infrequent (rare) large losses (typically Deck B), is always disadvantageous [57]. Therefore, it is not possible to tell whether choices for this riskiest deck are driven by risk preference or lower sensitivity to the relative EV of the decks [27]. In our analyses, which control for the relative advantageousness of risky choices, we found that adolescents did not take more risks overall relative to adults; rather, risk taking depended on other problem-specific factors, and especially on rare-outcome favorability. If, as we have found, adolescent participants tend to underweight rare outcomes more than adults, this would explain increased risk taking in this rare-unfavorable decision context. Based on IGT results, some have suggested that adolescents have a “frequency bias”, such that they prefer options with infrequent losses [58], which may contribute to risk taking. The idea that adolescents experience exaggerated underweighting of rare outcomes in experience can also explain preferences for the deck with infrequent negative outcomes. However, the rare-underweighting explanation is more general than the frequency bias account because it can be extended to situations with either favorable or unfavorable rare outcomes.

During experience-based decision making, we found only subtle age differences in sampling, supporting the idea that adolescents and adults may seek information similarly through experience. A prior study using a similar experience paradigm found significantly lower sampling in adolescence, aligned with the notion that adolescents have a relatively higher tolerance for uncertainty [21]. However, the present age difference in sampling was only marginally significant. Further, as a manipulation check, we asked participants to sample from distributions and estimate the probabilities of those distributions (see S1 File). In this task, adolescents took more samples than adults and performed similarly to adults when estimating the sampled distributions. It is possible that when asked to make estimations adolescents can do so accurately, but that when rendering decisions without this explicit goal, they do not automatically make accurate probability estimations based on sampling. Paradigms that ask participants to estimate sampled probabilities intermittently throughout experience (e.g., [59]) would be helpful in clarifying the nuances of age differences in sampling and estimation.

In both description and experience formats, adolescents were less sensitive to EV than adults, although this difference was only marginal in experience. These results are aligned with prior literature suggesting that adolescents tend to make less advantageous choices than adults [26,28,60]. Moreover, the differential age patterns in pupil dilation with respect to EV provide further evidence that EV is considered differently in teens’ than adults’ decisions. However, this finding is counter to a prior study reporting that adolescents are more sensitive to EV in description than adults [29], and to other studies showing that there is no difference between adolescents’ and adults’ advantageous decisions [22,30,31]. Notably, although relative EV in our decision problems systematically varied, the EV difference between risky and safe options was small and not always obvious. EV differences between risky and safe options in the present study had an absolute value that ranged from 0 to 3.4 points, and the highest-magnitude point outcome possible in one of our decision problems was 44 points. Thus, in our study, the largest EV difference in a choice problem was less than 8% of the highest possible point outcome. In contrast, in [29] where adolescents were more sensitive to EV than adults, EV differences between risky and safe options ranged from $0 to $7.50, while the largest-magnitude outcome was $20. Therefore, EV differences represented up to 38% of the largest magnitude point outcome, perhaps making the higher-EV option easier to discern than in the present study.

It is also worth noting another difference between our paradigm and others that test EV sensitivity in developmental samples. In the present study, the safe option was different on each trial. In contrast, most other paradigms that test EV sensitivity are framed as a decision to play or pass on risky gambles, and the safe option (i.e., “passing”) yields the same number of points on each trial (e.g., [22,25,30–32]). Past research has demonstrated different patterns of attention and choice when options vary across trials compared to when one option remains constant across trials (e.g., [61]). Perhaps adolescents’ lower sensitivity to EV in the present study was the product of difficulty in determining the higher-EV option, paired with increased attentional demands required to track two options that varied on each trial. It is also possible that the difficulty of identifying the higher-EV option made age differences more pronounced. Alternatively, if participants realized that the EV of risky and safe options was similar, perhaps other relevant contextual factors (e.g., favorability of rare and frequent outcomes) were prioritized in adolescents’ decision making.

Several limitations in the current study should be highlighted and addressed in future research. Although our sample size was determined a priori based on the prior literature at the time data was collected, we acknowledge that the study may not have been adequately powered to detect small effects. Thus, additional replications are necessary using similar experimental approaches inspired by the adult decision-making literature, but with larger sample sizes to ensure that these findings are generalizable. Larger sample sizes will also facilitate the examination of individual differences in risk taking as a function of factors like sex, education level, and IQ, which we did not test in the present study. Additionally, although we preliminarily tested for linear age effects in risk taking and PT-consistent choice, these effects did not reach significance, perhaps due to a limited age range or sample size to detect such effects. Future studies designed to chart age patterns continuously can help us understand whether, and how, risk taking changes across adolescence into adulthood [62]. Furthermore, because our primary ambition was to conduct a traditional D-E gap experiment (e.g., [6]) with a developmental comparison, we only included problems with rare outcomes (e.g., including an option with 10% or 20% likelihood). Using this limited problem set required us to make inferences about probability weighting based on patterns of decision making rather than using computational modeling to estimate loss-aversion and probability-weighting parameters. Additionally, we defined rare outcomes in the same way as past studies of the D-E gap [6,7], as outcomes with less than or equal to 20% likelihood. It is important to note that this definition when conceptualized was somewhat arbitrary [6], but useful in improving the chances that the rare event would be sampled through experience. However, events people often consider to be rare (e.g., winning the lottery) have much lower probability than 20% or even 10%, which may limit the generalizability of our findings. Further, although our study did not include a condition with both descriptions and experienced information, such a condition would be helpful in gaining a better understanding of how both forms of information are integrated. It possible that when adolescents combine information from descriptions and experience, underweighting of rare outcomes in decision making is exaggerated. Additionally, because the trial structure differed between description and experience (e.g., sampling in experience took more time than rendering a choice in description), we were not able to compare process measures across description and experience. Despite these shortcomings, we hope the current study will inspire future developmental work to continue to incorporate ideas and approaches borrowed from the adult decision-making literature. For instance, future research would benefit from directly probing physiological differences across description and experience in developmental samples.

Together, the findings from this study highlight the utility of considering task format in conjunction with other contextual factors and age-dependent biases in understanding age differences in risky decision making. To return to the decision-making scenario in this article’s introduction, our results suggest that the adolescent deciding whether or not to drink at a party might underweight the rare possibility of getting caught in part based on their prior experience, and based on the descriptions of unfavorable rare outcomes that they may have learned from parents or in school. Although this finding might evoke a grim outlook on adolescent decision making (i.e., that adolescents will underweight the possible consequences of poor choices no matter the mode of information presentation), it is possible that descriptions can be helpful in emphasizing the positive aspects of prosocial risks [63]. Further, decisions in the real world are much more complex than those in our paradigm, typically involving both descriptions about and experiences with more than two options, with many possible consequences. Future work can build upon these findings to gain a more complete picture of when adolescents do–and do not–take more risks than adults.

## Supporting information

S1 File Supporting information. (DOCX)
