# Supplementary material for: Do adolescents always take more risks than adults? A within-subjects developmental study of context effects on decision making and processing
Source: PLoS One. 2021 Aug 2;16(8):e0255102. doi: 10.1371/journal.pone.0255102 (PMC8328301; doi:10.1371/journal.pone.0255102)
Supplement: S1 File — (DOCX) [file pone.0255102.s001.docx]

**Supporting Information**

*Table S1.* Full list of decision problems

*
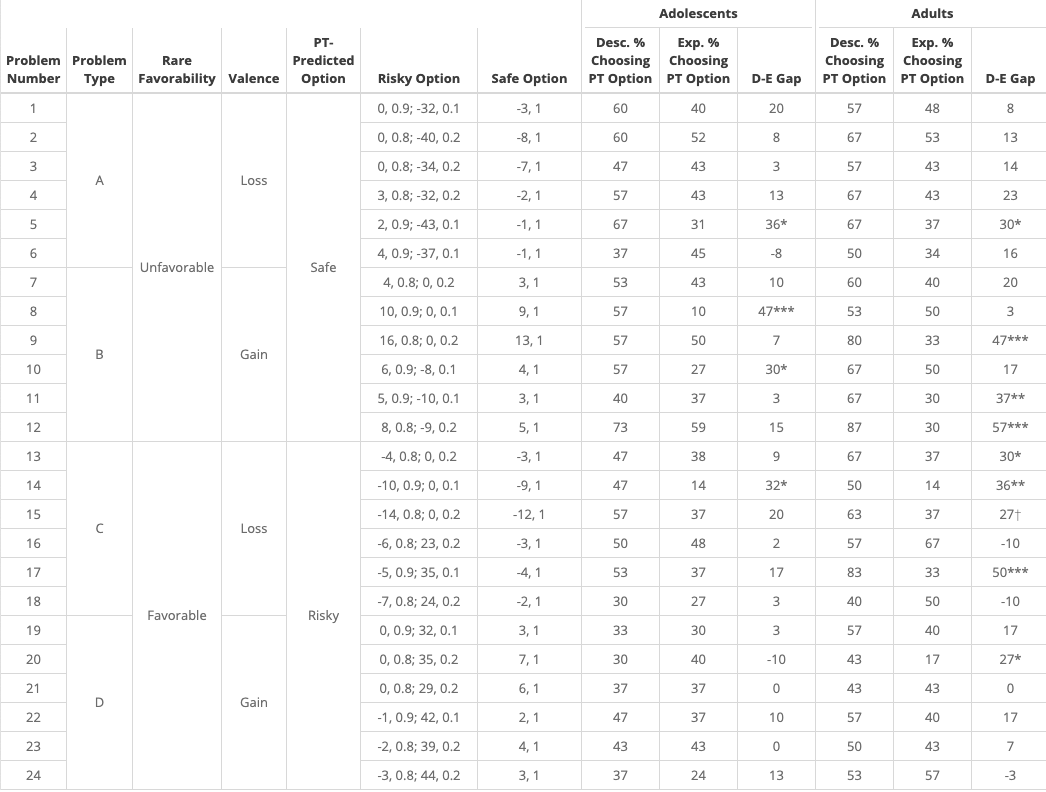
*

**Eye-tracking Preprocessing**

Eye tracking data were preprocessed using in-house MATLAB scripts. In preprocessing, we identified time points for which data were valid for at least one eye. If data were valid for both eyes we used the mean gaze position and pupil diameter. On the trials where data were valid for only one eye, we used data for the valid eye.

When cleaning looking time data, we removed very short fixations lasting less than 50ms that were unlikely to reflect attention to choice options. We then summed fixation duration toward risky and safe options. Finally, we computed the proportion of looking time at the risky option (versus the safe option), excluding fixations falling outside the risky and safe options (i.e., at other areas of the screen), and included this metric as an independent variable for a glmer regression to index gaze biases.

For pupil dilation analyses, we defined the baseline pupil diameter as the mean diameter in all valid samples in the 100ms preceding stimulus presentation. We computed percent change from this baseline value in the last 1/3 of looking time in the trial. Regressions including pupil dilation also included a variable indicating the proportion looking time at the brightest areas on the screen (areas with pie charts reflecting high probabilities, which were represented as white circles on a black background) to control for light-evoked pupil constriction. This value reflected the proportion of the total valid time looking in these bright areas relative to valid looking time anywhere on the screen during the last 1/3 of the trial.

**Additional Notes on Methodology**

An initial goal of the present study was to understand whether choice behavior differed between single- and mixed-valence problems. However, regression analyses including a binary variable indicating whether a problem was single or mixed domain, did not reveal any significant contrast effects (all ps>.1). Therefore, we did not include this variable in reported analyses.

Due to a coding error in the first 8 adolescent participants and the first adult participant, vertical displays showed the frequent value and probability on the left, and horizontal displays showed the frequent value on the top. Gaze data did not differ between these participants and the rest of the sample after the error was corrected, so all data were included in eye-tracking analyses.

**Risk Taking in Description and Experience**

In order to compare adolescent to adult risk taking in a way that is comparable that in prior developmental studies, we ran a 2 (format: description, experience) x 2 (age group: Adolescent, Adult) ANOVA. This analysis revealed that participants took marginally more risks in experience (*M*=48%) than description (*M*=46%; *F*(1,106)=2.82, *p*=.095, *η*^2^_p_=.03). There was neither a significant main effect of age group, nor a significant interaction between age and format (*F*s<1), consistent with our hypothesis that adolescents and adults take risks at similar rates in description (Table 1, H1.A.) but in contrast to the notion that adolescents take more risks in experience (H1.B.).

**Testing for Linear Age Effects**

We ran linear regressions to test whether risk taking, or the proportion of PT-consistent choices, varied as a function of linear age in our adolescent sample. None of the regressions reached significance (Risk taking: Description: *b* = 0.006, *t*(25) = 0.38, *p* = .705, *f*^2^ = .01; Experience: *b* = -0.029, *t*(25) = -1.67, *p* = .11, *f*^2^ =.11; PT-consistent choices: Description: *b* = -0.003, *t*(28) = -0.121, *p* = .905, *f*^2^ =.001; Experience: *b* = -0.03, *t*(25) = -1.26, *p* = .22, *f*^2^ =.06).

**Description Response Time**

Because response time (RT) was skewed in both adolescent and adult age groups, we log-transformed RT for analyses (but summary statistics were reverse-transformed into RT for interpretability). Log RT did not significantly differ by age group (*Mdn*_adolescents_=5772.45; *Mdn*_adults_=6403.51, *W*=451.00, *p*=.994, *r*=.00). We ran a lmer regression to test whether RT varied by problem type, and whether any problem-type differences depended on age group. Results are displayed in Fig S1. Participants responded significantly more quickly to gain than loss problems. Additionally, participants responded marginally faster when the rare outcome was unfavorable than when it was favorable. We also found converging evidence that Log RT did not vary by age group. Follow-up regressions revealed that none of the problem-level variables interacted with age group (EV: *b* = -0.002, *t*(1404) = -.13, *p* = .893; Rare Favorability: *b* = 0.02, *t*(1404) =.45, *p* = .655; Valence: *b* = 0.03, *t*(1404) = .75, *p* = .453).


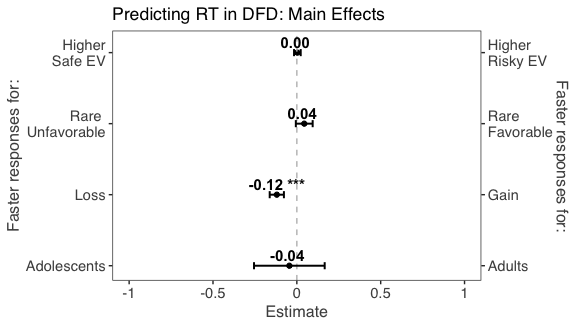

Fig S1. Results from lmer regression predicting RT by problem type and age group.

**Sampling Error**

In free-sampling experience paradigms, participants are unlikely to experience the exact underlying probability distribution. Importantly, less sampling can also lead to a less accurate representation of the underlying distribution (i.e., increased sampling bias; Hertwig et al., 2004). We examined whether the degree of deviation from the underlying probability distribution differs as a function of age group. For each problem and each participant, we quantified sampling error as the unsigned difference between the proportion of experienced samples yielding the rare outcome and the true underlying probability of sampling the rare outcome. For example, if the participant encountered the rare outcome in 2 of 8 samples (25%), but the true probability likelihood of the rare outcome was 10%, then a difference of 15% (25%-10%) was calculated as the sampling error for that trial. Although there were age differences in the number of samples taken, sampling error did not differ significantly between adolescents and adults (*M*_adolescents_=13.80%, *M*_adults_=12.85%, *t*(58)=1.14, *p*=.26, *r*=.15). Another way to characterize sampling error is to compute the percent of problems for which all outcomes were experienced. In other words, we can ask how often participants observed the risky frequent outcome, the risky rare outcome, and the safe outcome through sampling, and whether that differed by age. We found that adolescents experienced all outcomes on 61% of trials, while adults experienced all outcomes on 62% of trials. This difference was not significant (*t*(57)=0.38, *p*=.71, *r*=.05), so despite the overall age difference in sampling, adolescents and adults had similar experience with sampled outcomes before rendering their decisions.

**Task Order**

We ran a repeated-measures ANOVA to test whether the proportion of PT-consistent choices in description or experience varied as a function of the counterbalanced first task. There were no main (*F*(1,114) = 0.07, *p* = .797, *η*^2^_p_=0) or interactive (*F*(1,114) = 0.03, *p* = .337, *η*^2^_p_=.01) effects of the task completed first on the proportion of PT-consistent choices in either format.

**Comprehension Questions**

Participants were presented with the following three comprehension questions at the end of the experiment to exclude out participants who may not have understood or been paying attention to instructions:

1. True or false: You had to sample each sampling machine at the beginning of the experiment 5 times

2. How many points did you start the experiment with?

a) 25

b) 50

c) 100

d) 200

3. How will your monetary reward be determined?

a) From a running total of all the points you won/lost during all of the games

b) From a randomly drawn game

c) From the combined total of 5 randomly drawn sampling machine outcomes
